# Pilot cadaveric study on the feasibility of cricothyroidotomy and the associated complications in 30 cats

**DOI:** 10.3389/fvets.2024.1365780

**Published:** 2024-04-08

**Authors:** Julia A. Delle Cave, Samuel R. Larcheveque, Edouard Martin, Elizabeth O’Toole

**Affiliations:** Faculté de Médecine Vétérinaire, Université de Montréal, Montreal, QC, Canada

**Keywords:** cricothyroidotomy, CICO, airway obstruction, difficult airway, feline, cricothyrotomy

## Abstract

**Objectives:**

The study’s primary goal was to assess the feasibility of the cricothyroidotomy technique (CTT) in cats and evaluate its success rate (i.e., secure airway access). Secondary outcomes were the assessment of the subjective difficulty of airway access based on body score condition and weight. Further secondary outcomes consisted of procedural time and scoring of associated complications. The current study hypothesized that the CTT procedure would provide secure airway access with a reasonable success rate.

**Materials and methods:**

A prospective experimental study assessing the performance of CTT and associated complications was conducted on 30 feline cadavers. A procedural datasheet was completed to subjectively grade difficulty of landmark palpation, guide placement and tube placement and expected success of the procedure. A dissection was then performed post-procedure by a blinded observer to evaluate for any associated damages.

**Results:**

CTT was successful in securing an airway in 100% of the cats. The time to completion of the CTT was rapid, with a median time of 49 s (ranging from 31 to 90 s) for securing an airway. Of importance, this procedure was judged to be overall easy (median “ease of procedure score” of 7/10; ranging from 3 to 10) by the experimenters. The post-procedural lesion rate was elevated (76.7%) in this population of cats, though based on the lesion scores, was deemed mild in 73.9% of the cases.

**Clinical significance:**

CTT warrants consideration as the primary option for emergency front-of-neck airway access for cats although further studies are necessary.

## 1 Introduction

The Difficult Airway Society defines a “cannot intubate, cannot oxygenate” (CICO) event as a respiratory distress event caused by an upper airway obstruction not responsive to conventional oxygen supplementation or conventional airway management (i.e., tracheal intubation) (1). This is a potentially fatal emergency that requires efficient, rapid, and successful access to a secure airway. Human medical guidelines on the management of difficult airways recommend that an emergency procedure to gain front-of-neck access should be performed if intubation is impossible and oxygenation / ventilation of the patient is not adequate (1, 2). Cricothyroidotomy (also termed cricothyrotomy, CTT) is recommended in Human guidelines (1–3) and has been proven to be the fastest and most reliable method of securing an airway in the emergency setting and is associated with fewer complications than temporary tracheostomies (TT) in human medicine (4). A number of CTT techniques, including open surgical techniques, scalpel-bougie or modified Seldinger techniques using various commercial kits and percutaneous needle technique, have been described in human patients with various success rates and insertion times (5–7) although the surgical techniques tend to be more successful (6, 8).

CICO events are rare in veterinary medicine hence the scarce literature and guidelines available regarding their management and most of the reference textbooks still recommend a temporary tracheostomy as the “go-to” step if oro-tracheal intubation is deemed impossible (9, 10). A recent brief review published in 2022 underlined the need for guidelines when facing a CICO event in veterinary medicine (11). There is little mention of the use of a surgical tube CTT in the veterinary literature despite it being the current standard in the 2015 Difficult Airway Society guidelines in human medicine (1). This procedure has begun to gain interest in veterinary medicine as indicated by recent studies in canines (12–16). A recent study on dog cadavers evaluated the feasibility of a cricothyrotomy for emergency front-of-neck airway access and demonstrated that the procedure time was significantly faster when compared to a tracheostomy (12). CTT has also been described in recent studies of prehospital advanced airway management in military working dogs (13–15). Finally, an isolated case-report reported the use of elective cricothyrotomy in a live dog (16). However, to the authors’ knowledge, no literature is currently available on the use of CTT in cats.

A prospective experimental study assessing the performance of CTT and associated complications was conducted on feline cadavers. The study’s primary goal was to assess the feasibility of the CTT technique in cats and evaluate its success rate (i.e., secure airway access). Secondary outcomes were: assessment of the difficulty of airway access based on body score condition (BCS) and bodyweight. Further secondary outcomes consisted of procedural time and scoring of associated complications. The current study hypothesized that the CTT procedure would provide secure airway access with a reasonable success rate (i.e., greater than 90% success rate in adequate placement of the tube in the trachea).

## 2 Materials and methods

30 cadavers were prospectively enrolled between October 2021 and February 2022 into the study at the veterinary teaching hospital of the University of Montreal. The ethics committee was consulted and it concluded that a review of the study’s protocol was not required as the procedures were performed on cadavers donated to the teaching institution. Cats were included if they were either euthanized or died at the teaching institution, the owner’s permission for cadaveric donation had been obtained and the procedure was completed within a 2-h timeframe from the death. Exclusion criteria included: cats less than 2 kg, less than 4 months of age, inability to complete the procedure within the specified timeframe (out of hours euthanasia or body donation or evaluators unavailable in the clinics), or the presence of overt signs of disease or trauma to the frontal anatomy of the neck (i.e., mass, trauma, wounds).

All CTT procedures were performed by one of three previously trained experimenters (One second-year American college of veterinary emergency and critical care resident, one emergency clinician and one emergency and surgery specialty intern). All the experimenters had performed more than 3 CTT on dog cadavers and 3 CTT on cat cadavers prior to the study. All 3 evaluators had been trained by a board-certified surgeon to evaluate for lesions or damage to the anatomic structures that potentially could occur during this procedure before commencing the study.

**Figure 1:**
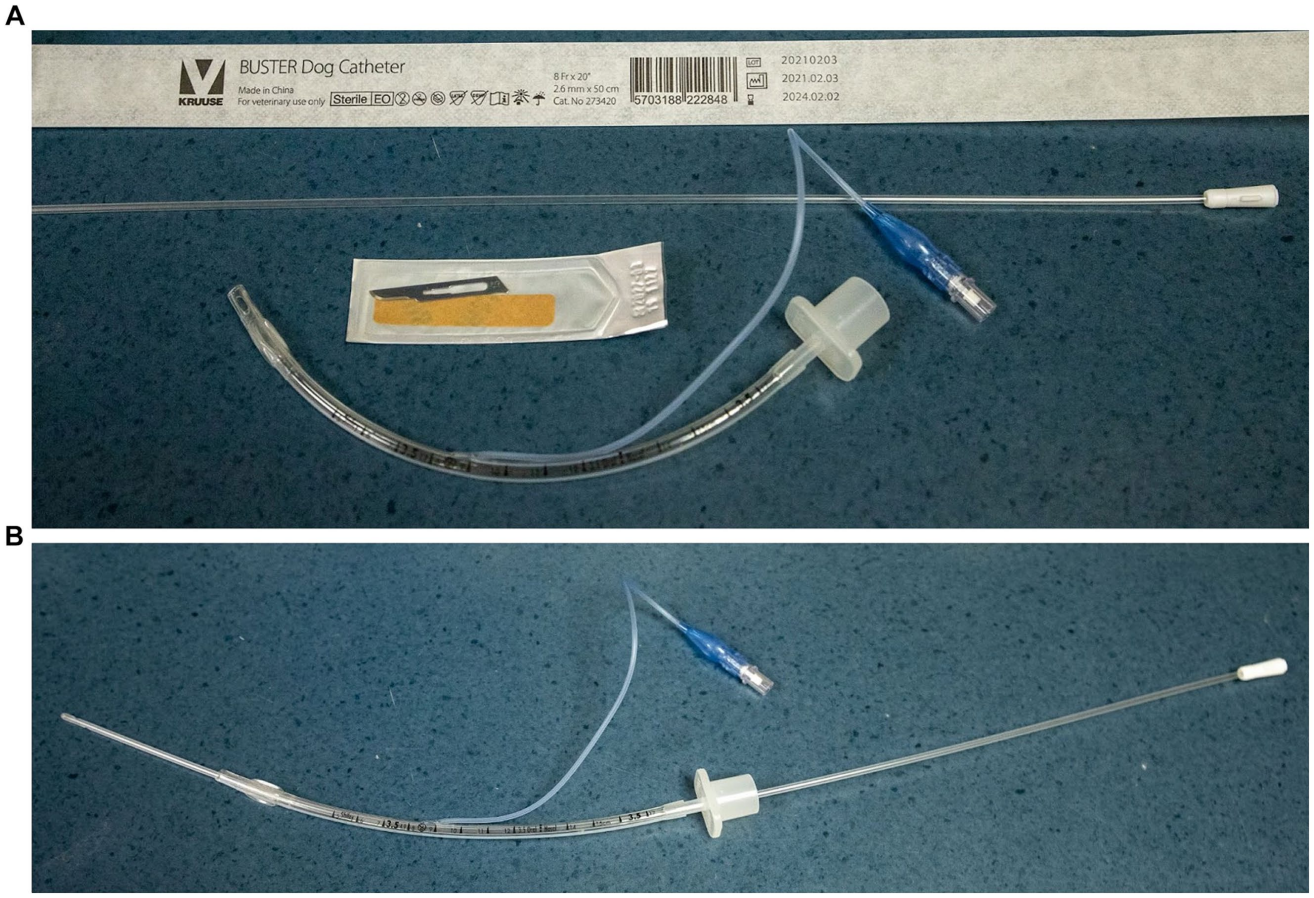
**(A)** Equipment necessary to perform CTT laid out next to the patient [50 cm rigid 8 French polypropylene dog urinary catheter*, a cuffed 3.5 French 16 cm rigid endotracheal tube (ETT)**, and a #15 scalpel blade***]. **(B)** Urinary catheter pre-placed in the 3.5 French endotracheal tube.

Each cadaver was placed in dorsal recumbency with a towel between the cervical spine and table to provide optimal exposure to the neck anatomy. Instruments for the procedure were pre-prepared and included the following: a 50 cm rigid 2.6 mm (8 Fr) external diameter polypropylene dog urinary catheter,^1^ a cuffed 3.5 Fr 16 cm rigid endotracheal tube (ETT),^2^ and a #15 scalpel blade (Figure 1A).^3^ The polypropylene urinary catheter was pre-placed inside the ETT (Figure 1B). The CTT technique used was based on one described by Hardjo et al. on dogs and pigs (12, 17). Experimenters were allowed to palpate surface landmarks prior to beginning the procedure. The ventral neck area of each cadaver was rapidly clipped and the duration of shaving was recorded. Laryngeal identification was performed by tracing the index finger of the non-dominant hand along the midline of the ventral neck starting caudally and moving in a cranial direction until the cricoid cartilage was identified. The cricothyroid membrane (CTM) was then palpated as a small and soft depression immediately cranial to the cricoid cartilage. An incision through the skin and soft tissues overlying the CTM using a #15 scalpel blade was then performed and a stab incision through the CTM was done to gain access to the airway lumen. The 50-cm polypropylene catheter was immediately passed into the stab incision with the blade still *in situ*. Once the catheter had accessed the airway, the scalpel blade was removed and the ETT was inserted into the airway over the polypropylene catheter as a guide. The insertion time was recorded by an independent third party, not involved in either the procedure or the evaluation of the procedure, and was recorded from the first incision to the moment when the experimenter verbally indicated completion with the word “stop.”

Immediately after completion, the experimenter completed a procedural data sheet (Table 1), including information on the cat (identification, age, body weight, BCS, and cause of death), difficulty scores (palpation of anatomic landmarks, guide placement, ETT placement), subjective assessment of the success of the procedure (i.e., tip of the endotracheal tube lying within the airway) and any anticipated complications as well as an “ease of the procedure” score. BCS evaluations were based on previously published scores (18). Success and presence of complications were based on a binary scale (i.e., yes or no answer). The “ease of the procedure” score was subjectively graded from 1 to 10 (1 being an extremely difficult procedure and 10 being an extremely easy one) by the experimenter, similar to a previously described study (12).

**Table 1:** Procedural data sheet: this table summarizes basic descriptive data regarding the included feline cadavers.

Animal	
Animal identification	
Age (months or years old)	
Weight (kg)	
Body condition score (/9)	
Cause of death or euthanasia	
Procedure	
Shaving time (s)	
CTT time (s)	
Difficulty to palpate anatomic landmarks (1 – 3)	1: easy 2: moderate 3: difficult
Comments regarding anatomic landmarks palpation	
Difficulty of guide placement (1 – 3)	1: easy 2: moderate 3: difficult
Difficulty of ETT placement (1 – 3)	1: easy 2: moderate 3: difficult
Procedure expected to be successful (yes/no)	
Complications expected (yes/no)	
Comments regarding procedure/technique	
Ease of procedure score (1 – 10)	1: being extremely difficult/unable to perform 10: being extremely easy

(s), seconds. It allows for subjective evaluation of the procedure by the experimenter that performed the CTT, using subjective scores of ease for the various steps of the procedure as well as an overall subjective score of ease of the procedure along with the presence or absence of expected complications.

**Figure 2:**
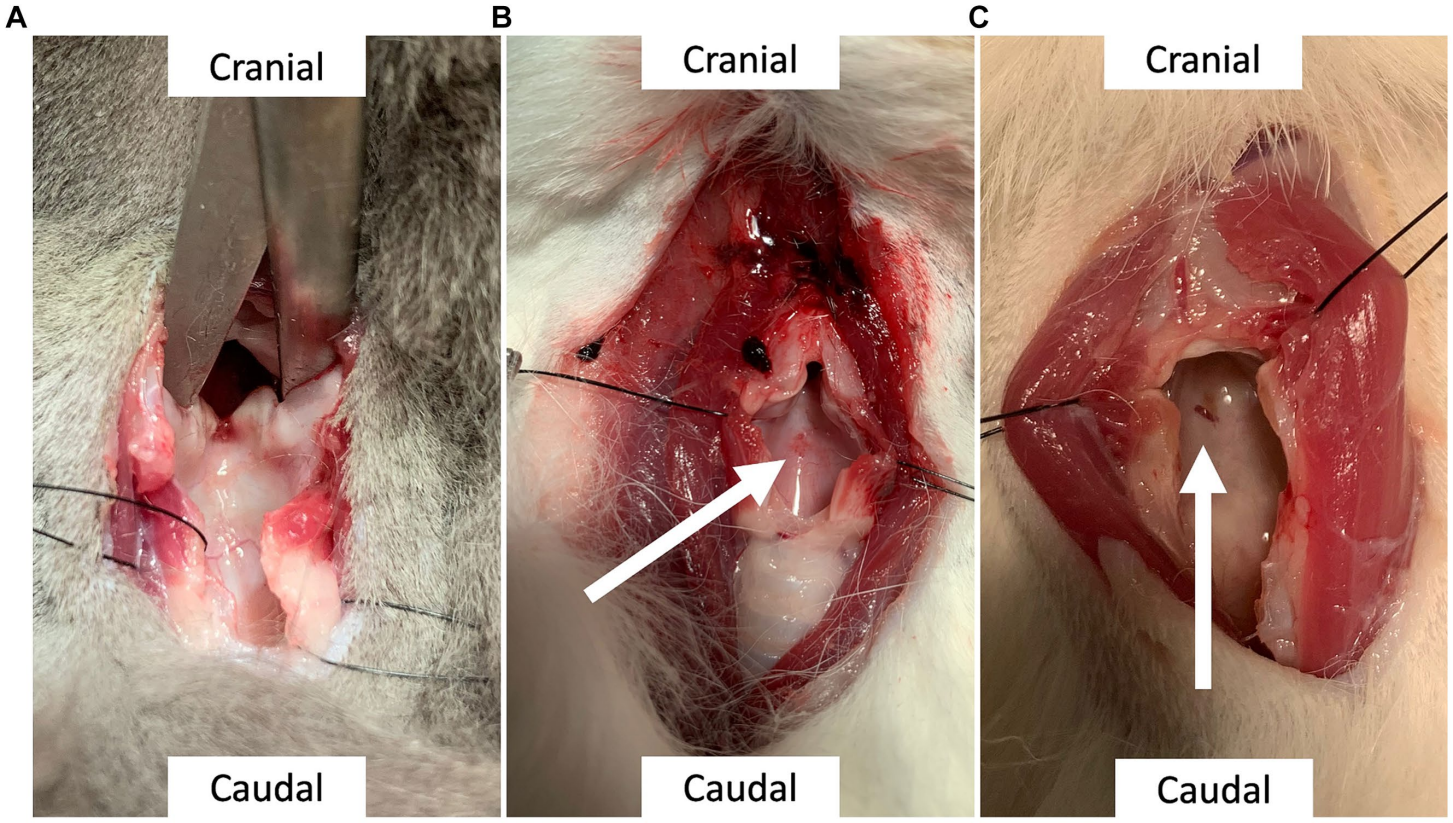
**(A)** Laryngeal lesion, grade 0: Lack of evidence of a lesion. **(B)** Laryngeal lesion, grade 1: Superficial injury on the luminal aspect of the dorsal cricoid cartilage, indicated by the arrow; measuring 0.3 mm. **(C)** Laryngeal lesion, grade 2: partial thickness mucosal laceration of the dorsal cricoid cartilage, indicated by the arrow; measuring less than 1 mm.

The cadavers were then examined and dissected by one of the other experimenters in the study within 12 h of the procedure. The post-procedure evaluator was not present during the procedure and was blinded to both the procedure and the procedural data sheet completed by the experimenter. A complete dissection was performed to confirm adequate tube placement within the airway and the presence of any tissue damage to the following areas: trachea, larynx, vocal fold, vessels, nerves, and esophagus. The evaluator then recorded the success of tube placement and tissue damage on the evaluator data sheet. A score was given for each injury type (Table 2). Injury scores were adapted from a similar veterinary study (12). Figures 2A–C show examples of grade 0, 1 and, 2 laryngeal lesions.

**Table 2:** Evaluator datasheet: the table describes, assesses and scores for any possible lesions or damages that occurred during the procedure.

Animal	
Animal identification	
Skin and subcutaneous incision	
Incision dimension (mm)	
Appropriate dimension (yes/no)	
Lesions	
Laryngeal lesions descriptions (0–3)	no gross damage minor damage: e.g., superficial scratches moderate damage, e.g., partial thickness mucosal laceration <5 mm Major damage, e.g., full-thickness tear, cartilage fracture
Tracheal lesions descriptions (0–3)	no gross damage minor damage, e.g., tracheal abrasions, scratches moderate damage, e.g., dorsal tracheal lesions, deformation of tracheal rings severe damage, e.g., full-thickness tracheal tear, tracheal ring fracture …
Vocal fold identified (yes/no)	
Vocal fold lesions (presence/absence)	
Muscular lesions description (0–2)	no gross damage minor damage, e.g., minor laceration of muscle <5 mm moderate damage, e.g., laceration of muscle >5 mm
Blood vessels lesions (presence/absence)	
Other lesions or comments	(ex. Esophageal tear, damage to associated nerves)
Overall procedure evaluation	
Overall damage score	no gross damage minor damage, e.g., tracheal abrasions, scratches, off-midline incision, minor laceration of muscle <5 mm moderate damage, e.g., dorsal tracheal lesions, partial thickness mucosal laceration <5 mm, laceration of muscle >5 mm, deformation of tracheal rings severe damage, e.g., full-thickness tracheal tear, tracheal ring or cricoid cartilage fracture, esophageal tear, incision between tracheal rings
Procedure success (yes/no)	

(mm), millimeters. The assessment and scoring were performed by the evaluator that performed the post-procedural dissection while being blinded to both the procedure and the procedural datasheet.

### 2.1 Statistical analysis

Descriptive statistical analysis was conducted on Excel version 16.69. Further statistical analysis was conducted in R Statistical program software 4.03 and G*Power version 3.1.9.7.

Data were summarized in accordance with their distribution and type, with normally distributed data presented as mean and standard deviation (SD), non-normal data as median and range, and where appropriate, categorical/binary data as proportion (%). Normality was assessed using a Shapiro–Wilk test. Correlations between procedure time and various variables (bodyweight, BCS) were assessed using either a Spearman correlation test or a Kendall correlation if the tested explicative variable was ordinal. The correlation between specific lesion score, their severity, and explicative values (body weight, BCS, procedure time) was assessed using generalized linear models. The effects of BCS and bodyweight on guide placement and the effects of the various explicative values (score of ease, BCS, procedure time, skin incision length, estimated complication, difficulty to palpate anatomical landmark, difficulty of guide placement, difficulty of endotracheal tube placement) on total damage score were assessed using a proportional odds model.

The significance level was set at 0.05.

## 3 Results

30 cadavers were prospectively enrolled. 36 cats met inclusion criteria but 6 cats were then excluded (2 because they did meet the sufficient weight cut-off, 1 because it was less than 4 months of age, and 3 because of the inability to complete the procedure within the specified timeframe).

The study population consisted of 24 domestic shorthair cats, 2 Persians, 1 of each of the following breeds: Highland lynx, Siamese, Bengal, and Sphinx. 14 cats were neutered males, 6 were intact males, 9 were neutered females and 1 was an intact female. The median age was 9 years old (ranged from 4 months old to 17 years old) and the mean body weight was 4.9 kg (SD 1.57; ranged from 2 to 7.1 kg) with a median BCS of 5/9 (ranging from 1 to 8). Reasons for death or euthanasia were various and included abdominal mass or effusion (6/30), trauma (4/30), respiratory condition (4/30), renal or urinary diseases (4/30), aortic thromboembolism (4/30), gastrointestinal condition (2/30), icterus (2/30), sepsis (1/30), neurological signs (1/30). The cause of death was unknown in 2 cases.

**Figure 3:**
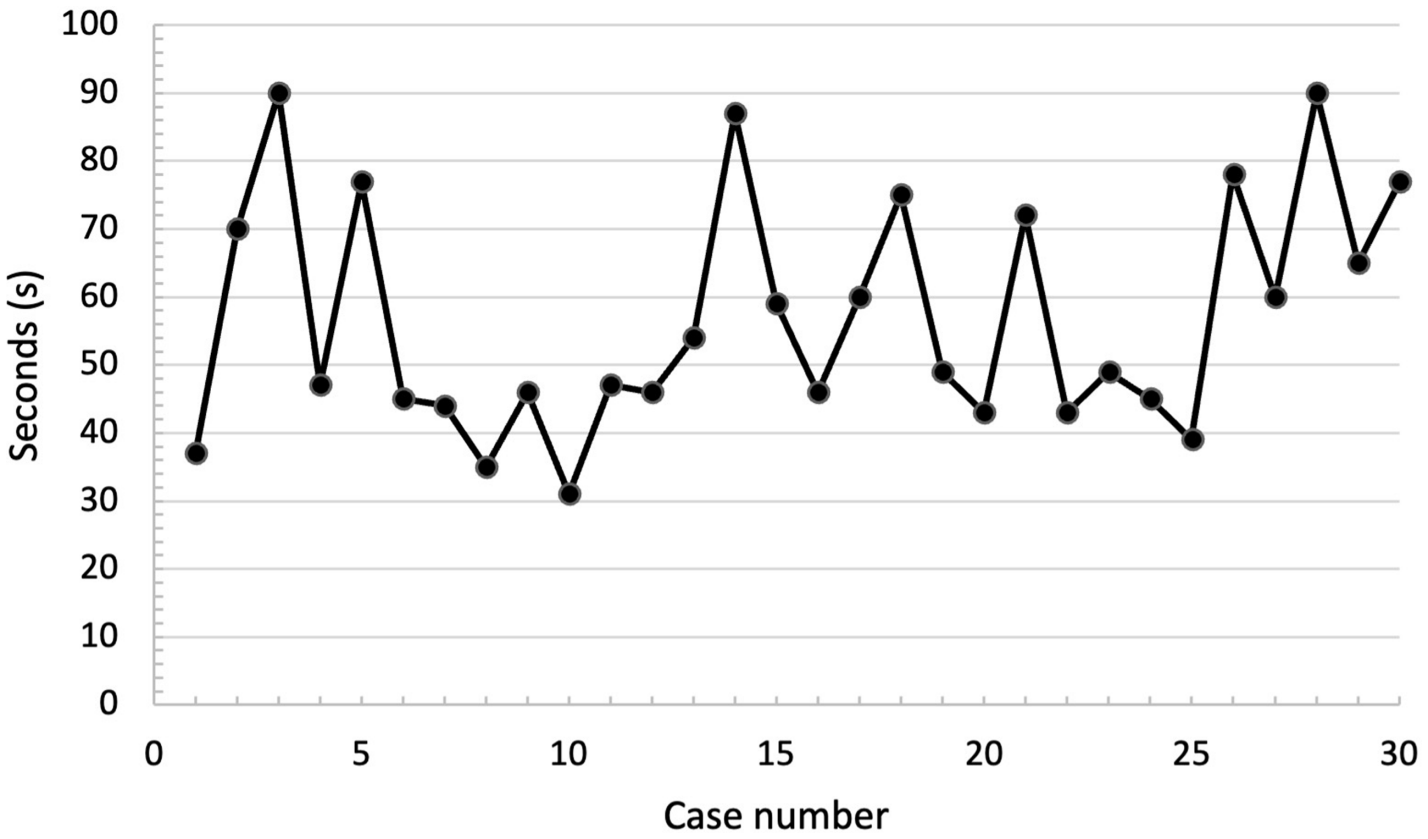
Procedural time, in seconds (s) according to case number (chronological order). No overt trend is identified.

**Figure 4:**
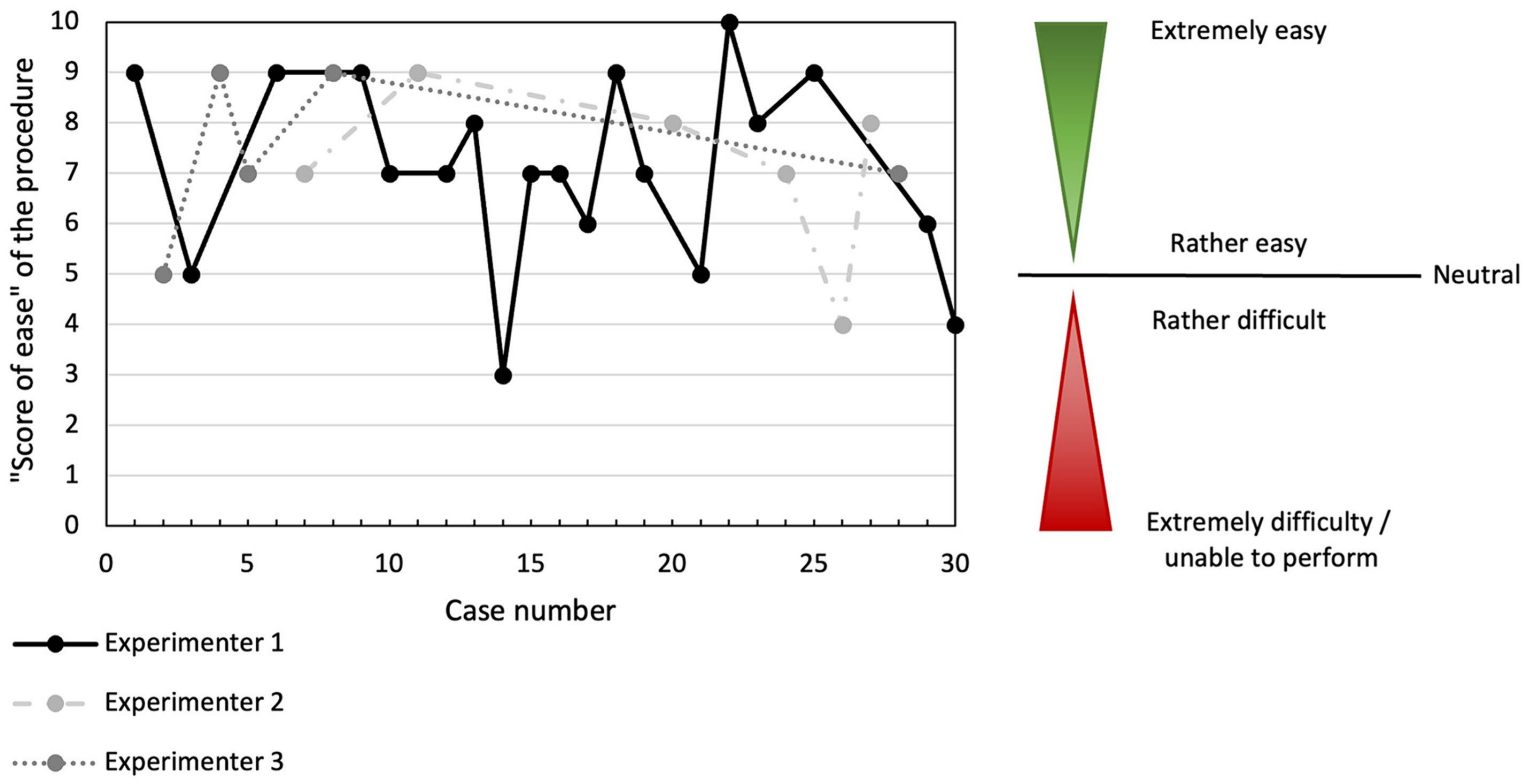
“Ease of the procedure” score according to case number (chronological order) and experimenter performing the CTT. “Ease of procedure score” were graded subjectively, as presented in Table 1. No overt trend is identified.

Median procedure time (including shaving) was 49 s (s) and ranged from 31 to 90 s (Figure 3). The procedure was judged to be successful by the experimenter in all cases and correct endotracheal tube placement was confirmed in all cases by evaluators (success rate of 100%). Anatomical landmarks were judged easy to palpate in 26/30 cats (86.7%); moderately difficult in 3/30 (10%) and difficult to palpate in 1/30 (3.3%). Experimenters subjectively assessed that guide placement was easy in 22/30 (73.3%), moderately difficult in 7/30 (23.3%), and difficult in 1/30 (3.3%) of the cats. Endotracheal tube placement was judged easy to perform in 8/30 (26.7%), moderately difficult in 14/30 (46.7%), and difficult in 8/30 (26.6%) of the cats. Those results are presented in Table 3. The median “ease of procedure” score was 7/10 (ranging from 3 to 10). Overall, the procedure was deemed rather difficult by the experimenters (score of ease less than 5) in only 3/30 (10%) of the cases (Figure 4).

**Table 3:** Difficulty scores: this table summarizes the results of post-procedural experimenter’s subjective assessment of the difficulty of the different steps of the procedure.

Procedure difficulty scores	Subjective grades	N (%)
Difficulty to palpate anatomic landmarks	1: Easy	26/30 (86.7%)
	2: Moderate	3/30 (10%)
	3: Difficult	1/30 (3.3%)
Difficulty of guide placement	1: Easy	22/30 (73.3%)
	2: Moderate	7/30 (23.3%)
	3: Difficult	1/30 (3.3%)
Difficulty of ETT placement	1: Easy	8/30 (26.7%)
	2: Moderate	14/30 (46.7%)
	3: Difficult	8/30 (26.7%)

The scoring system is the same as utilized in Table 1.

The mean length of the skin incision was 9.8 mm (SD = 2.97; ranged from 5 to 29 mm) and was deemed acceptable in 29/30 of the cases by the evaluators. It was judged too long in one case.

**Figure 5:**
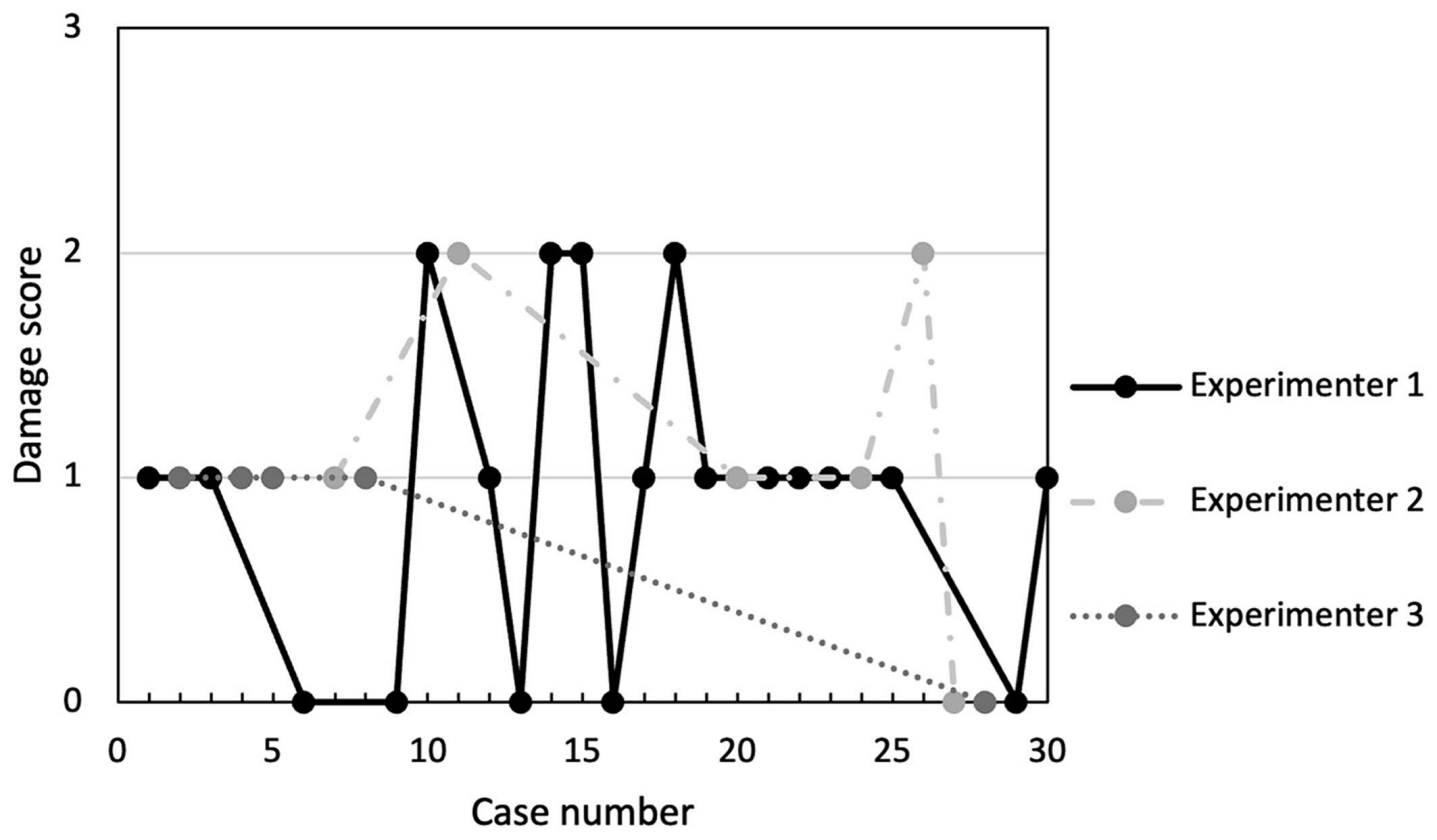
Damage score according to case number (chronological order) and experimenter performing the CTT. The scoring system used is presented in Table 2. No overt trend is identified.

The post-procedural evaluation revealed that 23/30 of cats (76.7%) had at least one lesion present. This consisted of a singular lesion in 10/23 cats (43.5%), which was a muscular lesion in 5/10 (50%) of them, and 13/23 cats (56.5%) had 2 distinct lesions. Details of the lesions and their severity are presented in Table 4. Vocal fold were only definitively identified in 26/30 of the cats. None of the 30 cats presented with indications of severe damage (e.g., full-thickness tracheal tear, tracheal ring or cartilage fracture, or esophageal tear). Lesions identified in the larynx were minor in 10/16 of the cases (62.5%) and moderate in 6/16 (37.5%) and implicated the arytenoid cartilage in 7/16 of the cases (43%). Example of laryngeal lesions of minor and moderate severity are presented in Figure 2. When “ease of procedure” score and damage scores were plotted overtime, there were no overt trend in damage severity with increasing experience for any of the 3 experimenters (Figures 4, 5).

**Table 4:** Post-procedural lesion severity scores: this table summarizes the results of post-procedural evaluator’s assessment of the various type and severity of lesions, after a dissection was performed, associated with the CTT procedure.

	Number of cats with lesions (%)	Scoring of the lesion (when applicable)	N (%)
Laryngeal lesion	16/30 (53.3%)	Grade 1	10/16 (62.5%)
		Grade 2	6/16 (37.5%)
		Grade 3	0
Tracheal lesion	1/30 (3.3%)	Grade 1	1/1 (100%)
		Grade 2	0
		Grade 3	0
Vocal fold lesion	2/26 (7.7%)		
Muscular lesion	17/30 (56.7%)	Grade 1	17/17 (100%)
		Grade 2	0
Blood vessels lesion	0/30 (0%)		
Other lesions (nerve damage, esophageal tear …)	0/30 (0%)		
Overall damage score	23/30 (76.7%)	Grade 1	17/23 (73.9%)
		Grade 2	6/23 (26.1%)
		Grade 3	0

The scoring system is the same as utilized in Table 2. The evaluator performing the post-procedural dissection was blinded to both the CTT procedure and the procedural datasheet.

Based on the procedural data sheets, experimenters suspected an associated lesion in 14/30 cases (46.7%). Given the results of the post-procedural analysis, the experimenters had a negative predictive value of 25% and a positive predictive value of 75% in predicting the absence or presence of a lesion.

The “ease of guide placement” score was not significantly associated with either the body weight or the BCS (*p*-value = 0.76 and 0.83, respectively). Statistical analysis demonstrated that the procedure time had a moderate positive correlation to body weight (Spearman correlation coefficient = 0.412, *p*-value = 0.026) and to BCS (Kendall correlation coefficient = 0.391, *p*-value = 0.005).

Regarding the presence and severity of specific lesions (laryngeal and muscular), the only significant correlation was between the BCS and the presence/absence of laryngeal lesions (Odds ratio = 1.14; *p*-value = 0.027), indicating that cats with a higher BCS were more likely to suffer from a laryngeal lesion. A trend was present between a longer procedure time and a higher risk of the presence of laryngeal lesion but it did not reach statistical significance (*p*-value = 0.059).

Multivariate models of damage score tested BCS, procedural time, “ease of procedure” score, skin incision length, expected complications, difficulty to palpate anatomical landmarks, difficulty in placing the guide, and difficulty in placing the endotracheal tube and demonstrated no significant correlations (Table 5).

**Table 5:** Proportional odds model to evaluate the effect of various variables: body score condition (BCS), procedural time, “ease of procedure” score, length of skin incision, suspected complications according to the experimenter, “difficulty to palpate anatomical landmarks” score, “difficulty of guide placement” score and “difficulty of endotracheal tube placement” score on the overall damage score.

Explanatory variable	Estimate	Standard error	*p*-value
BCS	0.43	0.25	0.08
Procedural time	−0.04	0.04	0.22
“Ease of procedure” score	−0.42	0.32	0.20
Length of skin incision	0.03	0.08	0.20
Expected complications	−0.05	0.81	0.95
“Difficulty to palpate anatomical landmarks” score	−1.54	1.31	0.24
“Difficulty of guide placement” score	1.18	0.78	0.13
“Difficulty of endotracheal placement” score	−0.31	0.57	0.59

Significance was set for a *p*-value ≤0.05. None of the results were significant.

## 4 Discussion

The results of this study indicated that the CTT method, as described by Hardjo et al. in a porcine and canine model (12, 17) could be utilized in cats and resulted in successful airway access in 100% of the cats. The time to completion of the CTT was rapid, with a median time of 49 s (ranging from 31 to 90 s) for securing an airway. Of importance, this procedure was judged to be overall easy (median “ease of procedure score” of 7/10; ranging from 3 to 10) by the experimenters in most of the cases. CTT was judged rather difficult (“ease of procedure” score less than 5) in only 3/30 cases. However, of significant note was that the post-procedural lesion rate was elevated (76.7%) though based on the lesion scores was deemed mild (73.9%) in this population of cats.

The procedural time in this study (median of 49 s) was similar to that previously reported in dogs (mean of 46.2 s) (12). In the current study, CTT was not compared to another procedure such as TT and, despite an extensive search, the authors were unable to confirm a procedural time in emergent situations for TT in cats from the literature, therefore a direct comparison cannot be made at this time. However, it seems highly probable that the CTT procedure would be more rapid in securing an airway than TT in cats, as it has been reported in human (19, 20) and canine (12) studies.

Facing a CICO situation, there are several factors to consider, the speed of securing an airway being of the utmost importance. However, the safety of the technique for the patient also has high priority. Although post-procedural lesions were frequently reported (complication rate of 76.7%) in this study, the majority of the lesions were mild (73.9%) and unlikely to be of significant clinical importance (i.e., muscular lacerations, small superficial lacerations of the laryngeal cartilage, superficial erosion of a tracheal ring). The CTT procedure in this population of cats did not result in any major complications such as transection of a laryngeal cartilage or fracture, similar to the reported findings in dogs (12). Reported complication rates during CTT are also quite high in the human literature and up to 32.1%, depending on the setting of the study (4, 21, 22) and up to 50% in the pediatric population (23). As cadavers were utilized in the current study and previous veterinary studies (12, 13), hemorrhagic complications cannot be evaluated and their incidence remains unknown. One study using this CTT technique in nine live pigs did not report any hemorrhagic complications (12, 13). One case report in feline medicine using CTM puncture for retrograde orotracheal intubation did report some bleeding during the puncture of the CTM (24). Although they have been reported in the human literature during CTT (25), they remain rare given the fibrous nature of the membrane (4, 22).

Contrary to adult patients, guidelines in pediatric patients regarding CICO events are scarce and the preferred procedure to secure an airway remains unclear (3, 26–28). One of the reported limitations of the CTT in the pediatric population is the difficulty of anatomical landmarks identification due to their size. Due to these limitations and concerns regarding the size of the cricothyroid membrane, surgical CTT is usually not recommended for children younger than 8–10 years old (26, 28). Those concerns could also be relevant to cats and small dogs. However, in the current study, the experimenters subjectively felt that landmark palpation was easy in 86.7% of the cases and despite the small size of the CTM in cats, tube placement was still successful in 100% of the case. Hence, some of the concerns in pediatric medicine may not translate to adult feline patients. We do note that the only cat for which an experimenter felt that landmarks palpation was difficult was a 4-month-old kitten and CTT may not be suitable for pediatric veterinary patients.

To date, there are no peer-review guidelines on airway management in small animals when facing a CICO event (11). While temporary tracheostomy tube placement is still considered the gold standard by multiple references (9, 10), there is little information currently available regarding successful outcomes or complication rates in an emergency setting in cats and several limitations should be considered. Most of the available studies report post-procedure complications such as tube dislodgment or occlusions (29, 30) and little information is available regarding complications that occur during the procedure. Similar to the CTT procedure in our study, the TT procedure would also necessarily be associated with a high lesion rate (especially muscular) during the dissection. Another limitation to the use of TT during an emergent situation is that the distance between the skin and trachea is influenced by body weight in cats (30), suggesting that the ease of a tracheostomy procedure may be negatively influenced by body weight. On the contrary, the distance between the skin and the CTM is not influenced by the body weight in cats (31), and the present study did not find any association between BCS or body weight and “ease of guide placement” score. However, there was a positive correlation between BCS and the procedural time and presence of a laryngeal lesion. Lastly, the cricothyroid membrane is located more cranially than the tracheal cartilage, reducing the incidence of complications such as pneumothorax or mediastinal damage in humans (31). Hence, even though the CTT complication rate was elevated in this study (76.7%), CTT could still be considered a reasonable alternative in extreme emergent situations to TT in cats.

Other factors to consider include (but are not limited to) the overall ease of the procedure, the facility of landmark location, and the low technical requirements to perform the CTT procedure. Required equipment for the presented technique of CTT is inexpensive and readily available when compared to the use of specific CTT kits or manufactured tracheostomy tubes (13).

Regarding the CTT procedure used in the current study, ETT placement was graded with more difficulty than guide placement (median score of 2 and 1, respectively) and the use of a non-cuffed or smaller tracheal tube may have made placement easier. The authors *a priori* choose the use of a 3.5 cuffed tube, to better represent a real-world scenario as this type of ETT tube is more commonly available in veterinary practice.

There are several important limitations to the current study. The most important is that it was likely underpowered to determine a significant difference in lesion severity for BCS, weight, and age in this population. *A posteriori* statistical analysis based on R2 calculation and Cohen effect evaluation allowed estimation of the number of subjects necessary to reach significance based on our results. To detect a significant association between bodyweight and presence / absence of either laryngeal or muscular lesion, respectively 42 and 14,487 cats would have had to be included.

Furthermore, the study population was not chosen at random and was dependent on donated cadavers.

Another significant limitation to this study is the subjective nature of the data presented regarding difficulty score and score of ease. In particular, there was a limited number of experimenters in the study, who had been previously trained in this procedure on both dogs and cats and were able to perform multiple CTT over a 5-months period. Outside of a study-setting, veterinarians are not expected to be performing CTT techniques as frequently, as CICO event are of rare incidence in veterinary medicine. Therefore the “ease of procedure scores” may not be generalizable to the wider practicing veterinary population. We felt that this data may still be interesting to be presented since in a veterinary former study, novice students preferred the CTT technique to the tracheostomy technique, although that was no significant difference in difficulty score between the two techniques (12).

Another limitation to that study is that it was not designed to compare CTT to tracheostomy in cats, as a comparison procedure was not performed. The authors decided not to perform a comparison study to allow for a greater number of cases included in this pilot feasibility study.

However, given the simplicity of the surgical technique, the rapidity of the procedure, and its overall success in obtaining a secure airway, the CTT is an attractive alternative to teach and to be aware of for the generalized veterinary population.

## 5 Conclusion

The results of this study demonstrated that the CTT using the method as described by Hardjo et al. (12, 17) was feasible in cats and that it was a rapid and highly successful technique to secure an airway in cat cadavers. While CICO situations are a rare occurrence in veterinary medicine, the obtaining of a secure airway in a timely fashion is of utmost importance. Given that it is a rare occurrence the use of a simple surgical technique, which is rapid with few potentially serious complications is of importance. This study, with these experimenters, has demonstrated that the CTT procedure in cats is feasible and was judged easy-to-perform with little serious post-procedural lesions. The lesion rate associated with the procedure was elevated (76.7%), but lesions were deemed mild in the majority of cases (73.9%). CTT should be considered a viable alternative to TT for emergency front-neck access, especially for veterinarians with limited surgical experience or those not already proficient in performing TTs, although further studies are required, including a study comparing success rate, procedure time, and damage score to tracheostomy as well as a study on live patients to evaluate for post-procedural long term complication such as laryngeal stenosis.

## Data availability statement

The raw data supporting the conclusions of this article will be made available by the authors, without undue reservation.

## Ethics statement

The animal studies were approved by Comité d’éthique de l’utilisation des animaux of Université de Montréal (CEUA). The studies were conducted in accordance with the local legislation and institutional requirements. Written informed consent was not obtained from the owners for the participation of their animals in this study because the study only included cats for which owners had previously agreed to body donation to the Université de Montréal (euthanasia/deceased form).

## Author contributions

JD: Conceptualization, Formal analysis, Investigation, Methodology, Writing – original draft. SL: Investigation, Writing – review & editing. EM: Conceptualization, Funding acquisition, Investigation, Writing – review & editing, Methodology. EO’T: Funding acquisition, Supervision, Writing – review & editing.

## Footnotes

^1^Buster, Kruuse, Denmark.

^2^Shiley, Medtronic, USA.

^3^Swann Morton, England.

## References

[1] Frerk C Mitchell VS McNarry AF Mendonca C Bhagrath R Patel A. Difficult airway society 2015 guidelines for management of unanticipated difficult intubation in adults. Br J Anaesth. (2015) 115:827–48. doi: 10.1093/bja/aev371, PMID: 26556848 PMC4650961

[2] Apfelbaum JL Hagberg CA Connis RT Abdelmalak BB Agarkar M Dutton RP. 2022 American Society of Anesthesiologists practice guidelines for management of the difficult airway. Anesthesiology. (2022) 136:31–81. doi: 10.1097/ALN.0000000000004002, PMID: 34762729

[3] Law JA Broemling N Cooper RM Drolet P Duggan LV Griesdale DE. The difficult airway with recommendations for management – part 1 – difficult tracheal intubation encountered in an unconscious/induced patient. Can J Anesth. (2013) 60:1089–118. doi: 10.1007/s12630-013-0019-3, PMID: 24132407 PMC3825644

[4] Zasso FB You-Ten KE Ryu M Losyeva K Tanwani J Siddiqui N. Complications of cricothyroidotomy versus tracheostomy in emergency surgical airway management: a systematic review. BMC Anesthesiol. (2020) 20:216. doi: 10.1186/s12871-020-01135-2, PMID: 32854626 PMC7450579

[5] Hill C Reardon R Joing S Falvey D Miner J. Cricothyrotomy technique using gum elastic bougie is faster than standard technique: a study of emergency medicine residents and medical students in an animal lab. J Soc Acad Emerg Med. (2010) 17:666–9. doi: 10.1111/j.1553-2712.2010.00753.x, PMID: 20491685

[6] Hubble MW Wilfong DA Brown LH Hertelendy A Benner RW. A meta-analysis of prehospital airway control techniques part II: alternative airway devices and cricothyrotomy success rates. Prehosp Emerg Care. (2010) 14:515–30. doi: 10.3109/10903127.2010.497903, PMID: 20809690

[7] Mabry RL Nichols MC Shiner DC Bolleter S Frankfurt A. A comparison of two open surgical cricothyroidotomy techniques by military medics using a cadaver model. Ann Emerg Med. (2014) 63:1–5. doi: 10.1016/j.annemergmed.2013.08.025, PMID: 24094476

[8] Morton S Avery P Kua J O’Meara M. Success rate of prehospital emergency front-of-neck access (FONA): a systematic review and meta-analysis. Br J Anaesth. (2023) 130:636–44. doi: 10.1016/j.bja.2023.01.022, PMID: 36858888 PMC10170392

[9] Fudge M. Endotracheal intubation and tracheostomy In: Silverstein DC Hopper K, editors. Small animal critical care medicine. Third ed. St. Louis: Missouri. Elsevier (2023). 1131–6.

[10] Mann FA. Temporary tracheostomy In: Creedon JMB Davis H, editors. Advanced monitoring and procedures for small animal emergency and critical care. Second ed. Hoboken, New Jersey: John Wiley & Sons (2023). 377–87.

[11] Hardjo S Goodwin W Haworth MD Purcell SL. A proposed guideline for performance of emergency surgical airways in small animals: analysis of five unsuccessfully managed cannot intubate, cannot oxygenate cases. Vet Sci. (2022) 9:39. doi: 10.3390/vetsci9020039, PMID: 35202292 PMC8879832

[12] Hardjo S Croton C Woldeyohannes S Purcell SL Haworth MD. Cricothyrotomy is faster than tracheostomy for emergency front-of-neck airway access in dogs. Front Vet Sci. (2021) 7:593687. doi: 10.3389/fvets.2020.593687, PMID: 33505998 PMC7829300

[13] Hardjo S Palmer L Haworth MD. Prehospital emergency cricothyrotomy in dogs part 1: experiences with commercial cricothyrotomy kits. Front Vet Sci. (2021) 8:705695. doi: 10.3389/fvets.2021.705695, PMID: 34604369 PMC8483268

[14] Hardjo S Haworth M Croton C Purcell S Goodwin W. Pre-hospital emergency cricothyrotomy in dogs part 2: airway sealing and ventilation using cricothyrotomy tubes. Front Vet Sci. (2023) 10:1129462. doi: 10.3389/fvets.2023.1129462, PMID: 36876002 PMC9981793

[15] Palmer LE. Concepts of prehospital advanced airway management in the operational K9: a focus on cricothyrotomy. J Spec Oper Med Peer Rev. (2019) 19:99–106. doi: 10.55460/KV13-RV6C, PMID: 30859536

[16] Hardjo S Nash K Day S Haworth M. Elective cricothyrotomy in a dog with transient laryngeal paralysis secondary to Australian paralysis tick (*Ixodes holocyclus*) envenomation. Aust Vet J. (2022) 100:440–5. doi: 10.1111/avj.13175, PMID: 35615962 PMC9546364

[17] Hardjo S Croton C Haworth MD. A pilot study evaluating the utility of a novel tube cricothyrotomy technique in providing ventilation in small animals using a live porcine model. Vet Med. (2019) 10:111–21. doi: 10.2147/VMRR.S216551, PMID: 31934552 PMC6711556

[18] Laflamme D. Development and validation of a body condition score system for cats: a clinical tool. Feline Pract. (1997) 25:13–8.

[19] Toye FJ Weinstein JD. Clinical experience with percutaneous tracheostomy and cricothyroidotomy in 100 patients. J Trauma. (1986) 26:1034–40. doi: 10.1097/00005373-198611000-00013, PMID: 3783779

[20] Groom P Schofield L Hettiarachchi N Pickard S Brown J Sandars J. Performance of emergency surgical front of neck airway access by head and neck surgeons, general surgeons, or anaesthetists: an in situ simulation study. Br J Anaesth. (2019) 123:696–703. doi: 10.1016/j.bja.2019.07.011, PMID: 31451190

[21] Scrase I Woollard M. Needle *vs* surgical cricothyroidotomy: a short cut to effective ventilation. Anaesthesia. (2006) 61:962–74. doi: 10.1111/j.1365-2044.2006.04755.x, PMID: 16978312

[22] DeVore EK Redmann A Howell R Khosla S. Best practices for emergency surgical airway: a systematic review. Laryngoscope Investig Otolaryngol. (2019) 4:602–8. doi: 10.1002/lio2.314, PMID: 31890877 PMC6929583

[23] Prunty SL Aranda-Palacios A Heard AM Chapman G Ramgolam A Hegarty M. The ‘Can’t intubate Can’t oxygenate’ scenario in pediatric anesthesia: a comparison of the Melker cricothyroidotomy kit with a scalpel bougie technique. Pediatr Anesth. (2015) 25:400–4. doi: 10.1111/pan.12565, PMID: 25370783

[24] Kim D Lee I Son WG. Modified retrograde intubation through the cricothyroid membrane in a cat with temporomandibular joint ankylosis. Vet. Med Sci. (2022) 8:1341–6. doi: 10.1002/vms3.789, PMID: 35384359 PMC9297776

[25] Boon JM Abrahams PH Meiring JH Welch T. Cricothyroidotomy: a clinical anatomy review. Clin Anat. (2004) 17:478–86. doi: 10.1002/ca.10231, PMID: 15300867

[26] Coté CJ Hartnick CJ. Pediatric transtracheal and cricothyrotomy airway devices for emergency use: which are appropriate for infants and children? Pediatr Anesth. (2009) 19:66–76. doi: 10.1111/j.1460-9592.2009.02996.x, PMID: 19572846

[27] Navsa N Tossel G Boon JM. Dimensions of the neonatal cricothyroid membrane – how feasible is a surgical cricothyroidotomy? Pediatr Anesth. (2005) 15:402–6. doi: 10.1111/j.1460-9592.2005.01470.x, PMID: 15828992

[28] Okada Y Ishii W Sato N Kotani H Iiduka R. Management of pediatric ‘cannot intubate, cannot oxygenate.’. Acute Med Surg. (2017) 4:462–6. doi: 10.1002/ams2.305, PMID: 29123910 PMC5649306

[29] Elmenhorst K Villedieu E Cantatore M Baines SJ. 70 complications and outcomes of temporary tracheostomy in 24 cats: a multicentric study from 2004-2020. BSAVA Congress Proceed. (2023). doi: 10.22233/9781913859152.34.7 41589552

[30] Guenther-Yenke CL Rozanski EA. Tracheostomy in cats: 23 cases (1998–2006). J Feline Med Surg. (2007) 9:451–7. doi: 10.1016/j.jfms.2007.06.002, PMID: 17693112 PMC10911508

[31] Hansen IK Eriksen T. Cricothyrotomy: possible first-choice emergency airway access for treatment of acute upper airway obstruction in dogs and cats. Vet Rec. (2014) 174:17–7. doi: 10.1136/vr.101244, PMID: 24277918

